# Supplementary figures and images for: Apoptotic cell clearance triggers epithelial fate reprogramming during prostate regression
Source: Cell Death Dis. 2026 Apr 10;17(1):462. doi: 10.1038/s41419-026-08565-9 (PMC13181137; doi:10.1038/s41419-026-08565-9)

Supplementary Figure 4A

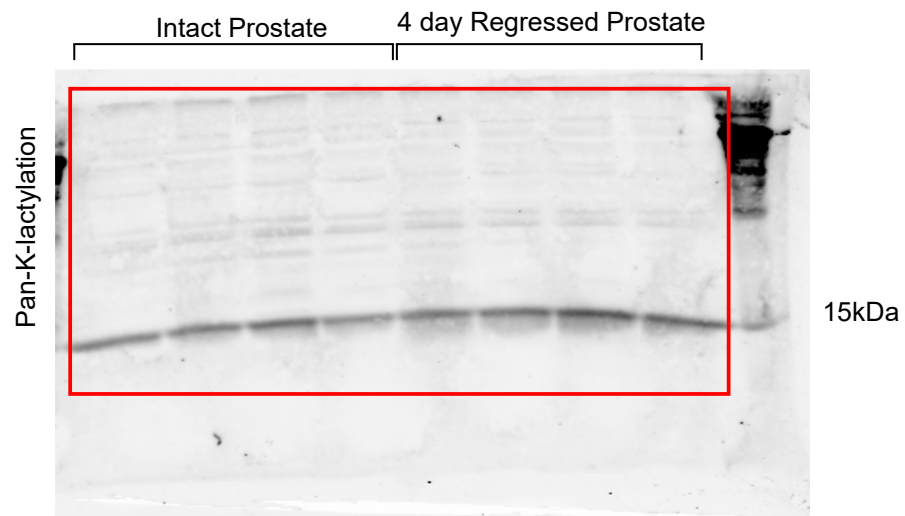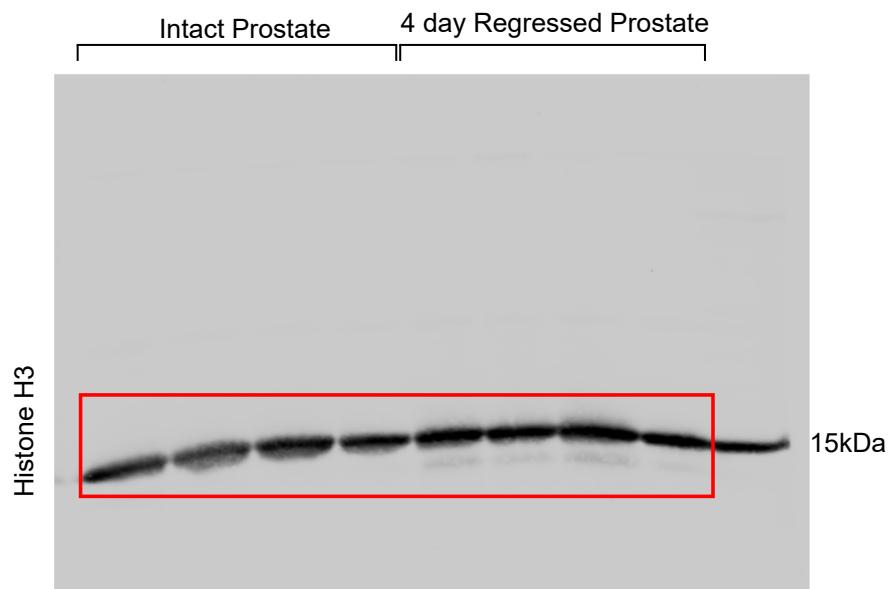

Supplement: Supplementary file 3 — Raw Western Blots [file 41419_2026_8565_MOESM3_ESM.pdf]
